# The ancestral haplotype markers HLA -A3 and B7 do not influence the likelihood of advanced hepatic fibrosis or cirrhosis in *HFE* hemochromatosis

**DOI:** 10.1038/s41598-023-35028-4

**Published:** 2023-05-13

**Authors:** John K. Olynyk, Richard Grainger, Helen Currie, Louise E. Ramm, Grant A. Ramm

**Affiliations:** 1 Medical School, Curtin University, Bentley, WA Australia; 2 Department of Gastroenterology, Fiona Stanley Fremantle Hospital Group, Murdoch, WA Australia; 3 QIMR-Berghofer Medical Research Institute, Herston, QLD Australia; 4 Faculty of Medicine, The University of Queensland, Herston, QLD Australia

**Keywords:** Liver cirrhosis, Liver fibrosis

## Abstract

Advanced hepatic fibrosis occurs in up to 25% of individuals with C282Y homozygous hemochromatosis. Our aim was to determine whether human leukocyte antigen (HLA)-A3 and B7 alleles act as genetic modifiers of the likelihood of advanced hepatic fibrosis. Between 1972 and 2013, 133 *HFE* C282Y homozygous individuals underwent clinical and biochemical evaluation, HLA typing, liver biopsy for fibrosis staging and phlebotomy treatment. Hepatic fibrosis was graded according to Scheuer as F0–2 (low grade hepatic fibrosis), F3–4 (advanced hepatic fibrosis), and F4 cirrhosis. We analysed associations between the severity of fibrosis and HLA-A3 homozygosity, heterozygosity or absence, with or without the presence of HLA-B7 using categorical analysis. The mean age of HLA-A3 homozygotes (n = 24), heterozygotes (n = 65) and HLA-A3 null individuals (n = 44) was 40 years. There were no significant differences between the groups for mean(± SEM) serum ferritin levels (1320 ± 296, 1217 ± 124, 1348 ± 188 $\upmu$g/L), hepatic iron concentration (178 ± 26, 213 ± 22, 199 ± 29 $\upmu$mol/g), mobilizable iron stores (9.9 ± 1.5, 9.5 ± 1.5, 11.5 ± 1.7 g iron removed via phlebotomy), frequency of advanced hepatic fibrosis (5/24[12%], 13/63[19%], 10/42[19%]) or cirrhosis (3/24[21%], 12/63[21%], 4/42[24%]), respectively. The presence or absence of HLA-B7 did not influence the outcome. Thus, HLA-A3 and HLA-B7 alleles are not associated with the risk of advanced hepatic fibrosis or cirrhosis in C282Y hemochromatosis.

## Introduction

Hemochromatosis is a common inherited iron overload disorder characterized by inappropriately increased iron absorption and excess iron accumulation in multiple organs, especially the liver and joints, eventually leading to organ dysfunction in some affected individuals^1–4^. It is most often due to a homozygous C282Y mutation in the Homeostatic iron regulator gene (*HFE)* affecting 1 in 150–200 people of European descent. Clinical sequelae occur much more commonly in men than women^1–4^.

Iron overload in *HFE* hemochromatosis results from a pathological decrease in the production of hepcidin, an important iron-regulating hormone that controls export of iron from reticuloendothelial cells and enterocytes into circulation^5–8^. Whilst the impaired production of hepcidin is known to be a direct result of the homozygous C282Y mutation and its effect on protein misfolding with the resultant absence of the HFE protein in the signaling pathway regulating hepcidin production, less than 40% of C282Y homozygotes develop hemochromatosis-associated morbidity and less than 25% develop advanced hepatic fibrosis^3^. In addition, up to one third of those with *HFE* hemochromatosis have ferritin levels within normal limits^4,9,10^. Crucially, the reason for incomplete penetrance of the C282Y mutation remains largely unexplained^11,12^, which has led to increased interest in identifying potential genetic and environmental modifiers that may influence the severity of phenotype expression in *HFE* hemochromatosis.

A number of environmental and genetic modifiers of phenotypic expression of *HFE* hemochromatosis have been described^11,12^. Prior to discovery of the *HFE* gene, hemochromatosis had been linked to human leukocyte antigen (HLA) markers, especially HLA-A3 and HLA-B7, and was shown to be transmitted as an autosomal recessive trait^13–16^. Variations in HLA haplotypes were also reported to influence the risk of severity of iron overload and phenotype of HFE hemochromatosis in multiple studies^17–22^. To interrogate this further, Barton et al. investigated a possible correlation between HLA-A3 and *HFE* hemochromatosis, to determine if heritable genetic modifiers of the C282Y homozygous mutation were linked with the haemochromatosis haplotype HLA-A3 and showed that most clinical manifestations of *HFE* hemochromatosis were not influenced by the presence or absence of HLA-A3^23^. Whilst they were able to assess the effect of HLA-A3 on noninvasive biomarker panels of advanced hepatic fibrosis in *HFE* hemochromatosis, they were unable to systematically evaluate the effect of HLA-A3 carriage on fibrosis stage due to the absence of liver biopsies in the majority of their published cohort. To clarify this uncertainty, we used a well-characterised cohort of *HFE* hemochromatosis individuals who had all undergone liver biopsy to stage hepatic fibrosis to elucidate whether HLA-A3 homozygosity, heterozygosity or its absence, with or without HLA-B7, was associated with the likelihood of advanced hepatic fibrosis.

## Methods

### Study population

**Figure 1:**
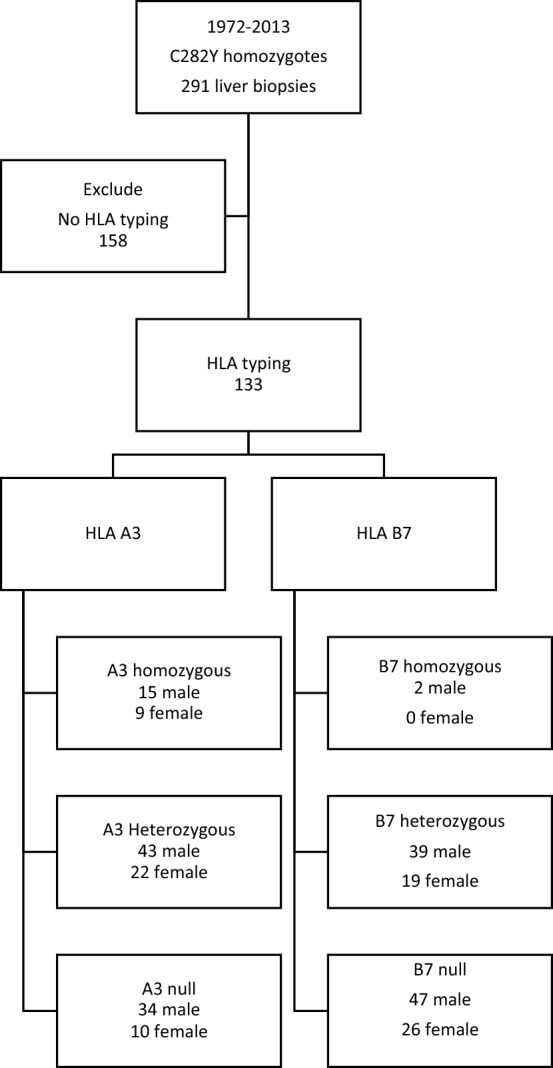
Subject recruitment for study inclusion.

A total of 291 subjects with confirmed C282Y homozygosity and concurrent liver biopsy were identified for the study. All had undergone liver biopsy at the Royal Brisbane and Women’s Hospital in Australia between 1972 and 2013. All subjects were offered liver biopsy as part of routine standard of care and baseline assessment and only those who declined biopsy did not receive one. Baseline demographic data, total number of venesections, alcohol consumption, biochemical results and liver biopsy histological assessments were available on all subjects. Exclusion criteria included age less than 16 years, other forms of chronic liver disease such as viral hepatitis, immune-mediated liver disease and metabolic liver disease. Subject age was defined as the age at the time of liver biopsy. Alcohol consumption was defined by the National Health and Medical Research Council of Australia as 1 standard drink containing 10 g of alcohol; safe recommended consumption levels being ≤ 210 g per week for males and ≤ 140 g per week for females. The hepatic iron concentration (HIC) was determined using atomic absorption spectrophotometry on fresh liver biopsy specimens^24^. Fibrosis staging was performed by liver histopathologists with expertise in HFE hemochromatosis using paraffin-embedded sections, stained with haematoxylin and eosin and Perls’ Prussian blue method. Fibrosis stage was classified according to Scheuer staging system: F0–no fibrosis, F1–mild fibrosis with enlarged portal tracts, F2–moderate periportal and portal-portal septa but intact architecture, F3–severe fibrosis with architectural distortion; and F4–cirrhosis with architectural distortion^25^. For the purposes of this study, subjects with hepatic fibrosis stages F3 and F4 were combined and termed ‘advanced fibrosis’. All subjects were untreated at the time of study inclusion, and weekly phlebotomy was performed until a serum ferritin level less than 100 µg/L was achieved. Continued venesection requirement and frequency was then determined by the treating physician. Mobilizable iron was calculated using total number of venesections required (one unit = 500 mL) to obtain a serum ferritin below 100 µg/L multiplied by 250 mg of iron. Of the 291 subjects that underwent liver biopsy, 133 also had HLA typing information available and constituted the final cohort for the study (Fig. 1). There were no differences in age, alcohol consumption, HIC, or liver fibrosis staging between those who either did or did not undergo HLA typing. HLA typing was performed using standard microlymphocytic reaction by the Immunology Laboratory at Princess Alexander Hospital in Brisbane^20^. These studies were approved by the Human Research Ethics Committees of the Royal Brisbane and Women’s Hospital and the QIMR Berghofer Medical Research Institute, Brisbane, Australia and informed written consent was obtained at the time of entry into the study.

### Statistical analysis

All results are presented as mean ± standard error of the mean (SEM) unless otherwise stated. Liver biopsy served as the gold standard for diagnosis of advanced hepatic fibrosis. Continuous data were analysed using unpaired t-test. Categorical data were analyzed using Chi-square testing. All statistical tests were performed using GraphPad Prism 9.4 (GraphPad Software, version 9.5.1, www.graphpad.com). A P value < 0.05 was considered statistically significant.

### Statement on guidelines

This study is in compliance with the Australian Code for the Responsible Conduct of Research, 2018.

## Results

General characteristics of the 133 C282Y homozygotes are shown in Table 1. There were 24 HLA-A3 homozygotes (15 male, 9 female), 65 HLA-A3 heterozygotes (43 male, 22 female) and 44 individuals who were null for HLA-A3 (34 male, 10 female). The mean ages, TS, SF, alcohol consumption, HIC and mobilizable Fe stores for the HLA-A3 homozygotes, heterozygotes and null individuals were not significantly different at diagnosis (Table 1).

**Table 1:** Characteristics at diagnosis of 133 men and women with *HFE* C282Y homozygosity.

Phenotype	HLA-A3 homozygotes (n = 24)	HLA-A3 heterozygotes (n = 65)	No HLA-A3 (n = 44)
Male, female (n)	15, 9	43, 22	34, 10
Mean age (yrs, ± SEM)	39 ± 3	40 ± 2	40 ± 2
Mean TS (%, ± SEM)	77 ± 4	80 ± 2	80 ± 3
Mean SF (µg/L, ± SEM)	1320 ± 296	1217 ± 124	1348 ± 188
Alcohol (g/d, ± SEM)	30 ± 8	20 ± 4	39 ± 7
HIC (µmol/g, ± SEM)	178 ± 26	213 ± 22	199 ± 29
Mobilizable Fe (g, ± SEM)	9.9 ± 1.5	9.5 ± 1.5	11.5 ± 1.7

*Fe*, iron; *HIC*, hepatic iron concentration; *HLA*, human leukocyte antigen; *SEM*, standard error of the mean; *SF*, serum ferritin; *TS*, transferrin saturation.

In total, 23 subjects had cirrhosis (F4) whilst 28 were classified as advanced hepatic fibrosis (F3-4) (Table 2). Two individuals in each of the HLA-A3 heterozygote (2 of 65) and HLA-A3 null (2 of 44) categories did not have hepatic fibrosis staging information recorded. Similar proportions of HLA-A3 homozygotes (3 of 24, 12%), HLA-A3 heterozygotes (12 of 63, 19%) and HLA-A3 null individuals (8 of 42, 19%) had cirrhosis (Chi-square P = 0.751). Five of 24 (21%) HLA-A3 homozygotes, 13 of 63 (21%) HLA-A3 heterozygotes and 10 of 42 (24%) HLA-A3 null individuals had advanced hepatic fibrosis (Chi-square P = 0.922).

**Table 2:** Association between HLA-A3 typing and liver biopsy-staged fibrosis grading in men and women with *HFE* C282Y homozygosity.

Phenotype	HLA-A*03 homozygotes, (n = 24) n(%)	HLA-A*03 heterozygotes (n = 63) n(%)	No HLA-A*03, (n = 42) n(%)	Chi-square (P)
Cirrhosis (F4)	3 (12)	12 (19)	8 (19)	
No cirrhosis (F0-3)	21 (88)	51 (81)	34 (81)	0.751

Phenotype	HLA-A*03 homozygotes, (n = 24) n(%)	HLA-A*03 heterozygotes (n = 63)* n(%)	No HLA-A*03, (n = 42)* n(%)	Chi-square (P)
Advanced hepatic fibrosis (F3-4)	5 (21)	13 (21)	10 (24)	
No advanced hepatic fibrosis (F0-2)	19 (79)	50 (79)	32 (76)	0.922

*HLA*, human leukocyte antigen. Hepatic fibrosis staged according to Scheuer et al.^25^ *Two individuals in each of the HLA-A3 heterozygote (2 of 65) and no HLA-A3 (2 of 44) categories did not have hepatic fibrosis staging information recorded.

Sixty subjects had either HLA-B7 homozygosity or heterozygosity (Fig. 1) whilst 73 had a multitude of other HLA alleles, resulting in too few individuals in the non-HLA B7-containg groups to conduct meaningful analyses. Furthermore, of 89 HLA-A3 containing individuals (homozygotes and heterozygotes combined), 49 had both HLA-A3 and HLA-B7 whilst 40 possessed HLA-A3 but were negative for HLA-B7. Of the 60 HLA-B7 containing individuals, 15% had cirrhosis and 18% had advanced hepatic fibrosis. Of 73 individuals who did not possess HLA-B7, 21% had cirrhosis and 25% had advanced hepatic fibrosis. There were no statistically significant associations between the presence or absence of HLA-B7 and either cirrhosis (Chi-square P = 0.433) or advanced hepatic fibrosis (Chi-square P = 0.386). Of 49 individuals who had both HLA-A3 and HLA-B7, 14% had cirrhosis and 16% had advanced hepatic fibrosis. Of the remaining 40 individuals who had HLA-A3 but did not have HLA-B7, 21% had cirrhosis and 26% had advanced hepatic fibrosis. There were no statistically significant associations between the presence or absence of HLA-B7 in individuals who carried HLA-A3 in terms of the presence of cirrhosis (Chi-square P = 0.407) or advanced hepatic fibrosis (Chi-square P = 0.254).

## Discussion

The development of clinically significant iron overload in *HFE* C282Y homozygous haemochromatosis is highly variable, occurring in about 40 percent of individuals, and heavily influenced by genetic and environmental modifiers^3,10–12^. The most common clinical manifestation with the greatest potential to influence mortality is liver disease. Up to 25 percent of C282Y homozygous individuals can develop advanced hepatic fibrosis and are predisposed to development of complications related to cirrhosis or hepatocellular carcinoma^3^. There has been great interest in the role of the HLA-A3 containing ancestral haplotype, as a potential modifier of the clinical expressivity of haemochromatosis. A recent study by Barton and colleagues showed that there was no association between the HLA-A3 haplotype and many biochemical or clinical manifestations of haemochromatosis^23^. However, the conclusions related to the development advanced hepatic fibrosis or cirrhosis were limited to assessments based on retrospective calculations of non-invasive biomarker panels as only a minority of the study participants had liver biopsies available for their study^23^. The aim of our study was to determine whether the presence of the HLA-A3 allele influences the likelihood of liver biopsy-proven advanced hepatic fibrosis in a well-characterised cohort of subjects with C282Y homozygous haemochromatosis. We showed that the risk of advanced hepatic fibrosis or cirrhosis in the present cohort was not associated with HLA-A3 or HLA-B7 carriage.

Recent studies demonstrate a number of potential genetic modifiers of the development of iron overload or liver disease in *HFE* C282Y homozygotes, including the p.D519G variant of the glycerophosphate O-acyltransferase gene^26^. Variants in bone morphogenic protein (BMP)-2 may also modify the phenotype of *HFE* C282Y hemochromatosis and lead to high iron burden^27–29^. Furthermore, heterozygous mutations affecting the *BMP6* propeptide have been associated with an inappropriate reduction in hepcidin levels and mild iron overload in some but not all populations^30,31^. Gene variants of proprotein convertase subtilisin/kexin type 7 and patatin-like phospholipase domain-containing protein 3 also have been proposed as risk factors for liver disease and cirrhosis in subjects homozygous for the *HFE* C282Y mutation^32,33^.

The literature has been conflicted regarding the association between HLA status and the prevalence of advanced hepatic fibrosis. Cirrhosis in patients with hemochromatosis was significantly associated with HLA-A3 in some studies^21^ but not others^17^. The methodologies which have been used to ascertain advanced hepatic fibrosis or cirrhosis also have varied widely between studies, including imaging (computerised tomography scanning, abdominal ultrasonography or elastography), noninvasive biomarker panels (such as fibrosis-4 [FIB-4] or aspartate aminotransferase-to-platelet ratio index [APRI]) or liver biopsy^23^. While FIB-4 and APRI are useful noninvasive biochemical tools for identifying those at increased likelihood of advanced hepatic fibrosis^34,35^, they are not as accurate as liver biopsy in staging the severity of disease^35^. Our study confirms and extends the recent observations of Barton et al.^23^ In their study, Barton et al. evaluated 180 *HFE* C282Y homozygotes for liver fibrosis. Whilst all were able to be assessed using FIB-4 and APRI, only 61 had available liver biopsies for definitive assessment. In comparison, all subjects enrolled into our study were staged for hepatic fibrosis using liver biopsy. Our study demonstrated that HLA status does not have any statistically significant influence on the risk of advanced hepatic fibrosis or cirrhosis in Australian *HFE* hemochromatosis patients.

Potential limitations of our study may include sample size and heterogeneity of data available. Out of a total of 291 C282Y homozygous subjects, 158 were excluded due to absence of HLA typing leaving a residual cohort of 133 subjects for analysis. Nevertheless, the cohort is large for a liver biopsy-validated study on *HFE* hemochromatosis, well-characterized and contains detailed matching clinical, biochemical and histological information, with no demonstrable difference observed between HLA-A3 or HLA-B7 cohorts for mean age, gender distribution, alcohol consumption, serum iron biochemistry, HIC, and the prevalence of advanced hepatic fibrosis. The results of this study do not exclude the possibility that a locus linked to alleles other than HLA-A3 and HLA-B7 influences the likelihood of advanced hepatic fibrosis. Indeed, the complexity associated with evolutionary history, genetic drift and recombination in populations as they move throughout the world makes it highly likely that genetic and other HLA-related modifiers may be changing over time in C282Y hemochromatosis^16,21,36,37^.

We conclude that HLA-A3 and HLA-B7 cannot be used as predictive markers of liver biopsy-proven advanced hepatic fibrosis or cirrhosis in *HFE* C282Y homozygous hemochromatosis.

## Data Availability

The datasets generated during and/or analysed during the current study are available from the corresponding author on reasonable request.

## Abbreviations

APRI: Aspartate aminotransferase-to-platelet ratio index
FIB-4: Fibrosis-4
HFE: Haemostatic iron regulator gene
HIC: Hepatic iron concentration
HLA: Human leukocyte antigen
SEM: Standard error of the mean
